# Study of GNSS Loss of Lock Characteristics under Ionosphere Scintillation with GNSS Data at Weipa (Australia) During Solar Maximum Phase

**DOI:** 10.3390/s17102205

**Published:** 2017-09-25

**Authors:** Yang Liu, Lianjie Fu, Jinling Wang, Chunxi Zhang

**Affiliations:** 1 School of Instrumentation Science and Opto-electronics Engineering, Beihang University, Beijing 100191, China; lianjief@buaa.edu.cn (L.F.); zhangchunxi@buaa.edu.cn (C.Z.); 2 Abdus Salam International Centre for Theoretical Physics, Telecommunications/ICT for Development Laboratory, Trieste 34151, Italy; 3 Collaborative Innovation Center of Geospatial Technology, Wuhan 430000, China; 4 School of Civil and Environmental Engineering, University of New South Wales, Sydney, NSW 2052, Australia; jinling.wang@unsw.edu.au

**Keywords:** ionosphere scintillation, loss of lock, temporal and spatial characteristics, GNSS signals, solar maximum

## Abstract

One of the adverse impacts of scintillation on GNSS signals is the loss of lock status, which can lead to GNSS geometry and visibility reductions that compromise the accuracy and integrity of navigation performance. In this paper the loss of lock based on ionosphere scintillation in this solar maximum phase has been well investigated with respect to both temporal and spatial behaviors, based on GNSS observatory data collected at Weipa (Australia; geographic: 12.45° S, 130.95° E; geomagnetic: 21.79° S, 214.41° E) from 2011 to 2015. Experiments demonstrate that the percentage of occurrence of loss of lock events under ionosphere scintillation is closely related with solar activity and seasonal shifts. Loss of lock behaviors under ionosphere scintillation related to elevation and azimuth angles are statistically analyzed, with some distinct characteristics found. The influences of daytime scintillation and geomagnetic storms on loss of lock have also been discussed in details. The proposed work is valuable for a deeper understanding of theoretical mechanisms of—loss of lock under ionosphere scintillation in global regions, and provides a reference for GNSS applications in certain regions at Australian low latitudes.

## 1. Introduction

Ionosphere irregularities generated by inhomogeneous plasma bubbles can result in rapid fluctuations of radio wave amplitude and phase, referred to as ionosphere scintillation. This phenomenon usually occurs at equatorial and lower latitudes. Scintillation has a severe impact on Global Navigation Satellite System (GNSS) performance and applications. With increasing reliance on GNSS in life critical services, the study of the impact of scintillation has received a new impetus. One of the adverse impacts of scintillation on GNSS signals is the loss of lock (LoL) status, in addition to GNSS geometry and visibility reduction that compromise the accuracy and integrity of navigation performance.

Extensive studies have been carried out on ionosphere scintillation. The severest ionosphere scintillation usually occurs at low latitudes because of the effect of Rayleigh–Taylor instability around the equatorial region. Some sectors around Eastern Asia have been widely focused on and studied, taking geomagnetic activity influence into account [1]. It is widely accepted that scintillation is closely related to the behavior of plasma bubbles, which has been deeply investigated during storm time at low latitudes [2]. This study sheds light on the peculiarities of the regional characteristics of scintillation and considers that the physical mechanism may be determined by several factors. Several scintillation models have been proposed to estimate and compensate scintillation effects on GNSS navigation performances [3]. Researchers have studied the scintillation characteristics and scintillation effects on Global Positioning System (GPS), based on data sets collected in July and August 2012 in Hong Kong [4]. The influence of scintillation on GPS signals has also been studied in the European Arctic from 8 to 14 November 2004 [5]. In addition, researchers also investigated aviation GNSS performance under ionosphere scintillation conditions both at the equatorial region and high latitude [6,7,8]. Recently, with the increasing demand for navigation augmentation services for civil aviation, the ionosphere scintillation influence on SBAS has been specifically studied in the European Africa region [9].

Specifically, the influence of scintillation on GNSS tracking and positioning were under hot focus, as discussed in the South American equatorial sector by Brazil observations [10]. Other studies have paid much attention to statistical models of GNSS signal amplitude distortion by scintillation, such as the Hybrid Scintillation Propagation Model proposed by Nikolay et al. [11]. To better understand the influence of ionosphere scintillation on GNSS tracking characteristics, especially the loss of lock induced by distorted GNSS signals due to scintillation, more comprehensive studies are required based on extended period of observation and detailed investigations. Cycle slip, as one of the most immediate causes of loss of lock, was intensively researched in both single GNSS Precise Point Positioning (PPP) and INS integrated situations [12]. Researchers also proposed an algorithm to detect cycle slip under high ionosphere activity, such as forward and backward moving window averaging (FBMWA) and second order time difference phase ionosphere residual (STPIR) as Cai et al. have discussed [13].

The effect of cycle slips found to be severe enough to require consideration, has a close relationship with ionosphere plasma bubbles and ionosphere scintillation. One such case that occurred on 23 March 2004 was discussed through TEC monitoring [14]. Another case was discussed by Japanese GEONET data, demonstrating sharp GPS tracking break down [15]. A robust tracking loop was required to resist scintillation-induced loss of lock under severe scintillation when the amplitude scintillation index S4 was larger than 0.7 (S4 between 0.3 and 0.5, weak scintillation; S4 between 0.5 and 0.7, moderate scintillation) [16].

To step forward, several GPS and ionosphere scintillation monitoring networks have been implemented with specific scintillation monitoring receivers, collecting comprehensive yearly GPS TEC and scintillation index data for in depth studies. Researchers have investigated the impact of scintillation on GPS cycle slips and positioning at high latitudes under solar minimum conditions, using a dataset from Canadian High Arctic Ionosphere Network (CHAIN) [17]; and a similar study was implemented with a dataset from August 2010 to March 2013 collected at Alaska [18]. TEC disturbance and plasma bubbles were investigated by dense GPS and scintillation monitoring networks like Low-latitude Ionospheric Sensor Network (LISN) in South America [19]. The general statistic characteristics of GPS scintillation were described by studying a five year dataset provided by the Space Weather Service (SWS) of Australia [20].

The impact of ionosphere scintillation on GNSS performance does not end at cycle slips. Severe and continuous cycle slips lead to loss of lock. Loss of lock means the GNSS receiver no longer tracks the signal accurately; under such status navigation messages cannot be further decoded, leading to less visible satellites for positioning, thus degrading positioning accuracy. Considering the above studies, it is quite meaningful to find how GPS receiver suffers from loss of lock under ionosphere scintillation conditions.

To address this problem, this paper has investigated the scintillation-induced loss of lock with respect of temporal and spatial behaviors. The loss of lock statistics were obtained by comparison between theoretical satellite visible times and real satellite visible times. Theoretical satellite visible times were calculated by GPS orbit ephemeris, which was firstly filtered to eliminate satellite and clock range outliers, these outliers are due to wrong orbit ephemeris parameters, which can lead to large satellite orbit errors. Real satellite visible time was calculated using the observed scintillation datasets provided by the SWS of Australia. The observation time span was as long as five years, from 2011 to 2015. The datasets used to calculate real satellite visible time were processed to create a large data set.

This work aims at contributing to a better understanding of the scintillation-induced GPS loss of lock properties. To accomplish this task, temporal distribution features are firstly discussed to get a general picture of loss of lock. Statistics and their relationship with the solar sunspots index was displayed to demonstrate the solar activity reliance on scintillation-induced loss of lock. The daytime scintillation phenomenon and its relationship with loss of lock was further investigated by discriminating a correlation coefficient between daytime scintillation index and loss of lock time. The results show that the scintillation-induced loss of lock time is closely related to both amplitude and phase scintillation in the daytime case at the Weipa observation site during solar maximum phase. It is also found that the local time occurrence of severe loss of lock event changes with a seasonal variation each year. Scintillation-induced loss of lock event under geomagnetic storm conditions has also been discussed, taking the storm cases of this solar cycle into account.

In Section 2, a further description of ionosphere scintillation-induced loss of lock is provided as a baseline theory for the proposed study. The method of calculating temporal and spatial loss of lock percentage is also considered in this section. The proposed methods for data collection and preprocessing are involved. In Section 3, experiments are implemented and the results are discussed thoroughly based on the data preprocessed in Section 2. The conclusions and summaries of this study are given in Section 4.

## 2. Proposed Methods

### 2.1. Basic Theory of Loss of Lock under Ionosphere Scintillation

The radio frequency signals can be affected by scintillation both in amplitude and phase. Both phenomena can have a great impact on GNSS signals. In a GNSS receiver, the signal should be firstly acquired and tracked, then a navigation massage can be decoded. The tracking loop stability is generally evaluated by loss of lock time, which determines the GNSS tracking status when the satellite signal is visible at a mask-angle larger than 5 degrees. Theoretically satellite tracking time can be calculated by the geographic coordinates of satellites in orbit, and the location of the receiver. Thus given the ephemeris of satellite, the tracking time or theoretical locked time is determined.

However, for any GNSS receiver, the tracking loop is not completely stable. Any signal changes such as distortion, carrier phase disturbance and even strong multipath may lead to signal processing failure, resulting in tracking instability, and further loss of lock, as mentioned above. Loss of lock time is the time span of loss of lock, and in this work it is calculated from the difference between theoretical locked time and observed locked time for a given satellite. If the value of loss of lock time is larger than zero, it indicates a loss of lock status for the receiver during the given time span. In the proposed method, satellite ephemeris and clock failures were firstly detected using RINEX navigation files, then the loss of lock time due to scintillation was calculated and analyzed. The ionosphere scintillation monitor (ISM) usually produces direct value of amplitude and phase scintillation indices, which indicates intensity of amplitude and phase scintillation. Amplitude scintillation index S4 was calculated by:

(1) $$S_{4}=\sqrt{\frac{\langle I^{2}\rangle -{\langle I\rangle }^{2}}{{\langle I\rangle }^{2}}}$$

where *I* denotes the intensity of the signal power. The phase scintillation parameter was calculated by:

(2) $$\sigma_{\varphi }=\sqrt{\langle \phi^{2}\rangle -{\langle \phi \rangle }^{2}}$$

where *ϕ* is the detrended phase of the signal, and 〈 〉 indicates the average during a certain time interval. *σ_ϕ_* cannot be directly calculated from GPS data, and it is obtained from detrended phase observations. Loss of lock time is not usually presented as the output of ISM, but locked time for a given satellite can be easily acquired from ISM parameters, as Table 1 presents. In this work it is calculated from the difference between the theoretical locked time and the observed locked time.

The loss of lock is influenced by several factors, among which ionosphere scintillation can be one of the most frequent and consistent, since ionosphere scintillation can occur in low latitudes, high latitudes and sometimes even middle latitudes. The properties of ionosphere scintillation are influenced by solar and geomagnetic activities and local time in a complex way. With the change of the relative position between the Earth and the Sun, scintillation presents a seasonal variation with peaks during the equinox months, which has been widely studied and verified [21]. Thus, loss of lock under ionosphere scintillation can also be affected by the spatial distribution of ionosphere irregularities and clusters, and those related factors such as solar activities, geomagnetic disturbance and seasonal variations, are remaining in depth investigations to be implemented.

### 2.2. Data Representation

To calculate the scintillation-induced loss of lock time, both theoretical locked time and observed locked time are needed. The datasets processed in this study were collected from the World Data Center (WDC), which is organized by SWS. GPS RINEX data and scintillation data handled were measured by nine observatories around Australia, with different geographic locations, among which is the Weipa observatory (geographic: 12.45° S, 130.95° E; geomagnetic: 21.79° S, 214.41° E), located in low latitude, and where the south equatorial ionization anomaly (EIA) crest sometimes occurs at the same latitude. The data were observed from 2011 to 2015. GPS navigation data were recorded every two hours to mark satellite ephemeris, and orbit information was then calculated. With RINEX data theoretical satellite visible time can be further calculated. The scintillation data processed were recorded every 1 min in daily data files which contain nine basic parameters, as Table 1 shows, and Time, PRN, Azimuth and Elevation angles, and Scintillation Indices were all used in this work. Amplitude Scintillation Index S4 was considered as a metric, and it was classified into four categories of scintillation intensity. It was noted that to minimize the effect of multipath interference on the scintillation characteristics investigation, only satellites with mask angle above 30° were selected. However, without loss of generality, a basic mask angle above 5° was also considered to find the maximum theoretical locked time and real observed time, as recorded in the scintillation datasets.

### 2.3. Analysis Strategy

The focus of this study is to investigate the characteristics of scintillation-induced loss of lock time observed at low latitude in the Australia region. This work applies the Ground-based Scintillation Climatology (GBSC) method based on the work of Spogli et al. [22]. GBSC was first developed to map amplitude and phase scintillation occurrence at high latitude, moreover scintillation induced loss of lock occurrence has been of more concern herein, besides amplitude and phase scintillation occurrence. To complete the analysis, the percentage of loss of lock occurrence $P_{o}$ or loss of lock percentage is defined as:

(3) $$P_{o}=1-T_{obs}/T_{est}$$

where $T_{obs}$ is the observed time interval under scintillation for a given satellite. The time interval can be selected as one hour, a day or several days with similar temporal characteristics. $T_{est}$ is the estimated time of scintillation occurrence for a given satellite at a fixed time interval and is calculated theoretically with satellite orbit parameters and the location coordinates. To determine loss of lock occurrence induced by scintillation, $T_{obs}$ should be confined with amplitude and phase scintillation thresholds. Typical threshold values are 0.3 for amplitude scintillation and 0.2 for phase scintillation, to consider that ionosphere irregularities exist. To remove the problem of poor statistics, data selected in each time span should be confined above a given number, and statistical accuracy is defined as:

(4) $$R\%=100\times \sigma \left(N_{tot}\right)/N_{tot}$$

where $N_{tot}$ is the total sample for occurrence calculation under a given time interval, according to previous study, $\sigma \left(N_{tot}\right)$ is the standard deviation of number of data samples, R% between 2.5% and 10% can contribute to good statistical results. Data was collected using a GPS ionosphere scintillation monitor receiver, and SWS supplies 1 min scintillation indices data as explained in Table 1. The impact of solar activity on loss of lock time under ionosphere scintillation was investigated with data of the whole selected period. Moreover, seasonal characteristics of loss of lock under scintillation were also considered, the features of the equinox and solstice periods were respectively discussed for distinction. Spatial characteristics of scintillation-induced loss of lock were investigated, by mapping elevation and azimuth dependent loss of lock percentage under ionosphere scintillation.

## 3. Experiments

### 3.1. Temporal Statistics of Loss of Lock under Ionosphere Scintillation

**Figure 1:**
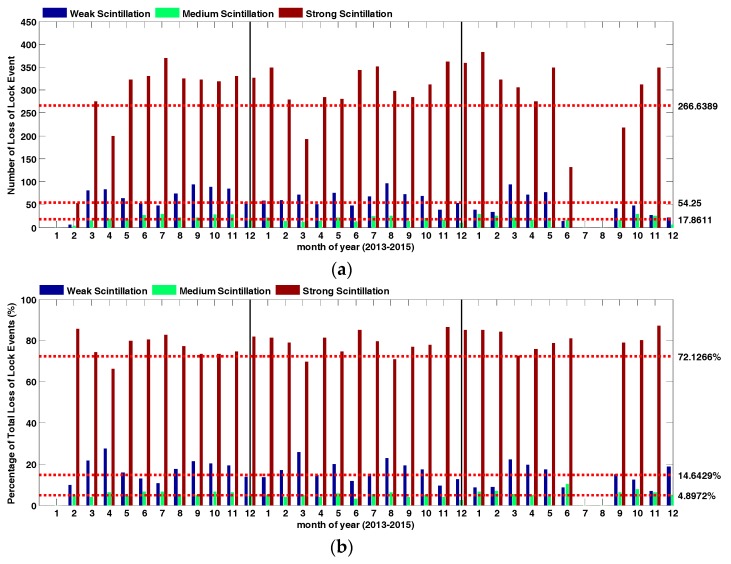
Temporal statistics of loss of lock events (**a**) and percentage; (**b**) occurred under ionosphere scintillation.

This section focuses on temporal statistics of loss of lock events occurred under ionosphere scintillation, and the dataset from 2013 to 2015 was investigated. Phase scintillation is mainly discussed, divided into three subclasses: weak phase scintillation (0.3 < *σ_ϕ_* < 0.5), moderate phase scintillation (0.5 < *σ_ϕ_* < 0.7) and strong phase scintillation (*σ_ϕ_* > 0.7). As Figure 1a,b show, most loss of lock events occurred under strong phase scintillation, with an average of about 267 cases per month, the percentage of loss of lock events was also dominant, with an average of about 72.13% per month. The results strongly support previous studies suggesting that phase scintillation plays a major role as a cause of loss of lock. Similar conclusions can also be reached for space-borne GPS receivers as [23] stated. The results also indicate that under strong phase scintillation, when the standard deviation of phase tracking error larger than 0.7 (more than 40°), even ISM GPS receivers cannot maintain stable tracking, and this should be addressed for further ISM receiver design. Another indication is that the intensity of phase scintillation under solar maximum can also be large even at low latitudes, where amplitude scintillation is dominant.

### 3.2. Seasonal Dependence of Loss of Lock under Ionosphere Scintillation

The seasonal characteristics of scintillation-induced loss of lock were calculated to investigate the relationship between seasonal variations and loss of lock time in accordance with different local times. The loss of lock occurrence percentage was defined as the percentage of loss of lock time interval during a season time span; four season time spans were selected as spring and autumn equinox, summer and winter solstice. The intensity of loss of lock occurrence percentage can reflect the influence from season changes on loss of lock time under ionosphere scintillation.

**Figure 2:**
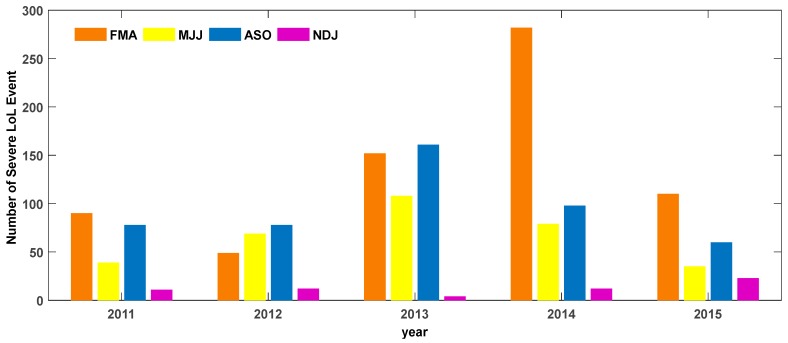
Seasonal statistics of loss of lock event by scintillation.

As shown in Figure 2, the loss of lock events are positively related with solar activities from 2011 to 2015, reaching a maximum in 2014, the year of larger solar activity. However, obvious differences were found for that for each year with different seasons. Generally the loss of lock behaved more actively at equinox season (FMA), reaching more than 150 cases at 2013. This was in consistence with the activities of ionosphere scintillation, which usually becomes more intensive at equinox, as referred previously [24]. Another apparent difference was the autumn equinox at 2014, when loss of lock cases reached its highest level, and more than 250 loss of lock events occurred during this time span. Further research may be conducted to analyze the reasons for the extremely loss of lock event in that season.

**Figure 3:**
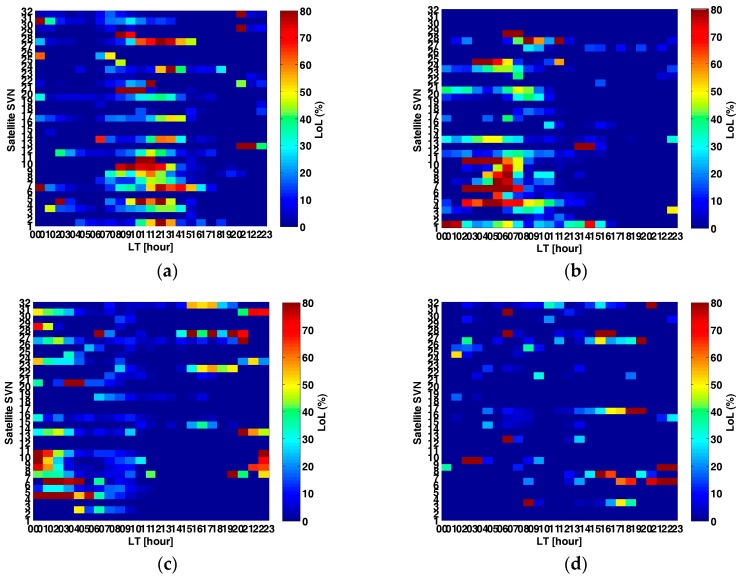
Seasonal statistics of loss of lock percentage by scintillation (from 2011 to 2015). (**a**) FMA; (**b**) MJJ; (**c**) ASO; and (**d**) NDJ.

To analyze the impact of seasonal transition on loss of lock, the correlation between local time, individual satellite and loss of lock occurrence percentage under ionosphere scintillation was further studied. It should be mentioned that the number of loss of lock events varied according to satellite PRN, due to the different ionosphere pierce points defined by different satellites. This can also reflect the regional conditions of ionosphere scintillation. The data were selected from 2011 to 2015, and each three months data were calculated to get loss of lock occurrence percentage for every one hour time span. A quite obvious feature found was that loss of lock occurrence percentage under ionosphere scintillation assembled within several hours, shifted according to the seasonal variations, as Figure 3 manifested. In Figure 3a satellite loss of lock occurrence percentage during the autumn equinox season (February, March, April) was mainly concentrated during daytime from LT 08:00 to LT 15:00; which was contrary to the occurrence of ionosphere scintillation in middle and low latitudes; the maximum loss of lock occurrence percentage went higher than 80% for PRN 9, PRN21 at LT 09:00 and PRN 10, PRN 28 from LT 10:00 to LT 11:00. Loss of lock occurrence percentage remained at high levels larger than 50% for PRN 4, PRN6 and PRN8 lasting from LT 09:00 to LT 13:00. The total time span was eight hours for the autumn equinox season. However, looking at Figure 3b that shows the winter solstice season (May, June, July), the loss of lock occurrence percentage “band” moved to LT 00:00 as beginning, and ended at LT 12:00, which means that the most severe loss of lock occurrence percentage can be found at midnight, and this is consistent with the common notion of scintillation occurring in middle and low latitudes. The maximum loss of lock occurrence percentage was higher than 80% for PRN 4, PRN 6 and PRN10 during LT 02:00 and LT 05:00, lasting for three or four hours. Similar higher loss of lock rate was found for PRN 24 and PRN 28, during LT 03:00 and LT 06:00. The total amount of loss of lock occurrence percentage above 20% was 13.2% for the autumn equinox and 13.8% for the winter solstice. Figure 3c shows the case for the spring equinox (August, September, and October). The loss of lock occurrence percentage occurred from LT 18:00 to LT 06:00, during the evening and midnight. As shown intensive loss of lock cases happened during midnight, from LT 00:00 to LT 03:00, for PRN 4, PRN 6 and PRN 10. Figure 3d has the lowest amount of loss of lock occurrence percentage above 20%, only 6.9%, indicating the summer solstice season case (November, December, and January). Also the time interval of loss of lock occurrence percentage was scattered, with two or three hours’ duration for the worst case. PRN 6, PRN 8 and PRN 17 suffered most from loss of lock under ionosphere scintillation.

In general, the seasonal variation of loss of lock occurrence percentage under scintillation is positively correlated with asymmetry equatorial ionization anomaly (EIA) proxy [25], and it is consistent with previous studies of the summer solstice case.

### 3.3. Daytime Scintillation and Its Effect on Loss of Lock under Ionosphere Scintillation

**Figure 4:**
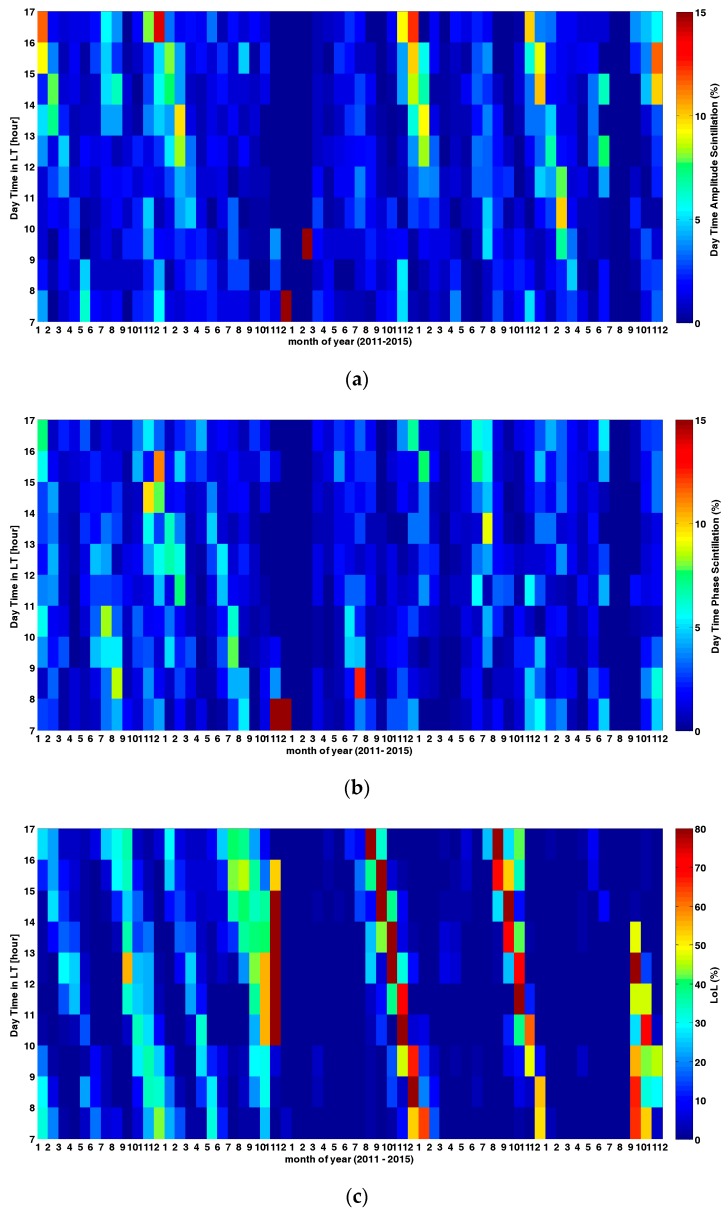
(**a**) Daytime amplitude scintillation percentage; (**b**) Daytime phase scintillation percentage; (**c**) Daytime loss of lock percentage by scintillation.

Ionosphere scintillation can also occur at daytime, though the occurrence percentage was relatively low compared to night side, according to its physical mechanism. Therefore, daytime scintillations were also found in both ground-based stations and space-borne observatories, with its unique cause compared with night time scintillations, as described by Basu and Dao [26,27]. Though most severe scintillation occurred at night side, the presence of daytime scintillation can’t be ignored, as stated by Seif [28]. In this section, daytime scintillation and its effects on loss of lock are discussed. It was found that loss of lock occurrence percentage during daytime scintillation varied according to the month. Figure 4 shows the monthly loss of lock occurrence percentage during daytime scintillation and its occurrence in local time, which moved in advance from LT 17:00 at January 2011 to LT 15:00 at February 2011, and so on. Loss of lock occurrence percentage during daytime scintillation reached a peak value during the spring equinox and summer solstice in 2013 and 2014, when the solar activities also reached a maximum. Note that the observatory is located in the south hemisphere, where the seasonal variations are opposite to its north counterpart. The frequent occurrence of loss of lock under daytime scintillation during the summer solstice goes against the traditional climatology of ionosphere scintillation caused by F region irregularities, this indicates that the cause of day time scintillation may be different.

**Figure 5:**
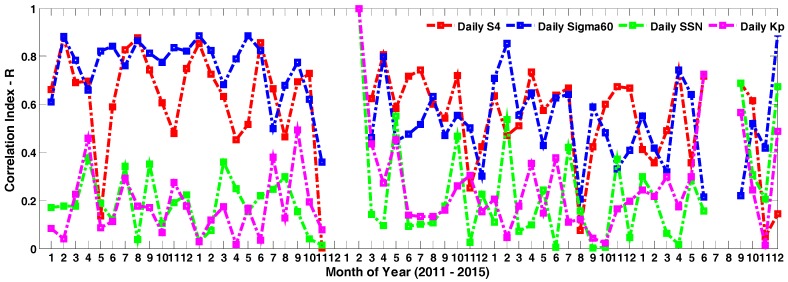
Correlation coefficients between daytime loss of lock percentage and daytime S4, daytime sigma60, daily SSN and daily Kp.

Moreover, the correlation coefficient was also calculated to verify this, as Figure 5 demonstrates. In fact, a close correlation can also be observed from Figure 4a,b, which described the daytime amplitude scintillation percentage and daytime phase scintillation percentage. However, as Figure 4c shows, daytime loss of lock occurrence percentage both correlated with daytime amplitude scintillation percentage and daytime phase scintillation percentage, and this was strongly improved by the correlation coefficient value represented by Figure 5, indicated in red and blue color. Similar correlation calculations have been used to analyze relationship between ROTI and scintillation index, as shown by Yang and Liu [29].

### 3.4. Spatial Distribution of Loss of Lock under Ionosphere Scintillation

**Figure 6:**
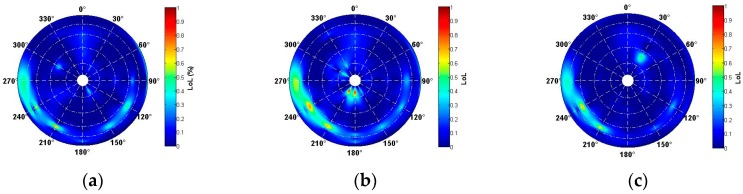
Spatial statistics of loss of lock percentage by scintillation. (**a**) 2013; (**b**) 2014; (**c**) 2015.

The above analysis mainly focus on the temporal distribution characteristics of the scintillation- induced loss of lock time calculated at the Weipa observatory during 2011 to 2015. In this subsection, the spatial distribution characteristics of scintillation were analyzed. The solar activity has a strong impact on loss of lock under scintillation, but it is still interesting to find that not each satellite will suffer from loss of lock even under severe scintillation during a given time span. Moreover, the intensity of loss of lock under scintillation was distinguished with ionosphere pierce points, which will be studied in this subsection. To further investigate this point, the intensity of loss of lock occurrence percentage under scintillation in different elevation and azimuth space regions were carefully calculated. Figure 6 demonstrates the spatial characteristics of loss of lock occurrence percentage at the solar maximum, from 2013 to 2015. On the premise that scintillation appears, the loss of lock occurrence percentage appearing in different regions were calculated and plotted in the sky charts by year. The charts were unfolded from a sky view, in which the center is the observatory local zenith and the circles around it are equal elevation contours. The lower bound of the elevation in the process was set to 30° to minimize multipath effects; therefore no results appears outside the 30° elevation contours.

It was shown that most severe loss of lock occurred at azimuth 270°, 240° and 210° for the three years, and most severe loss of lock occurrence percentage were observed at the year 2014 (Figure 6b), when solar activity reached its maximum value. It should be noted that loss of lock occurrence percentage under scintillation returned to its previous extent at the year 2015 (Figure 6c), though some small regions were shown to be dominant at azimuth 30° at high elevation angle, and this feature is worth further investigation. Severe loss of lock located between elevations 30° and 45° during the three years, where multipath effects on scintillation have been excluded. Similar calculations have done by Guo et al. [30].

**Figure 7:**
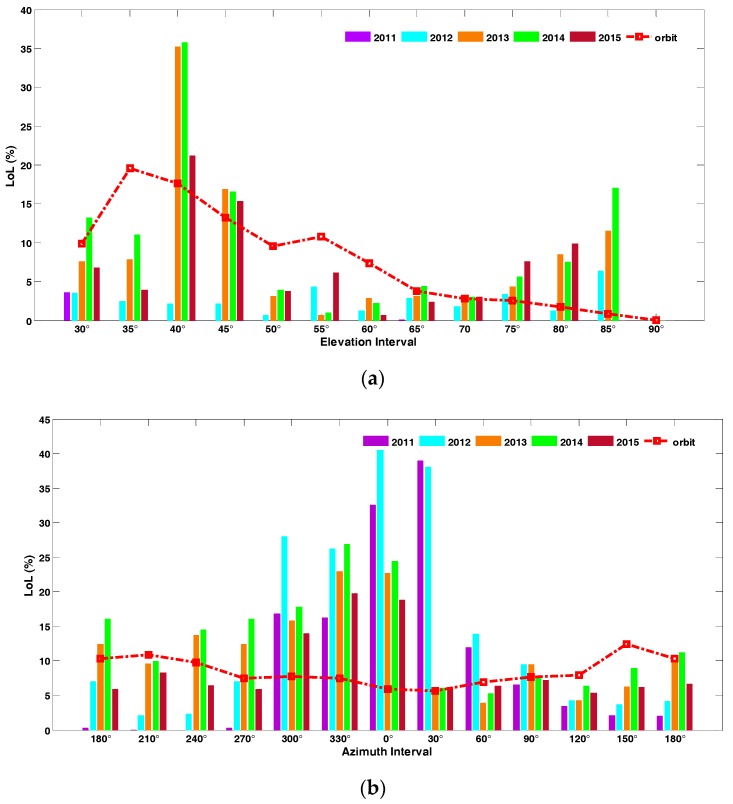
Loss of lock occurrence percentage towards elevation (**a**) and azimuth (**b**) from 2011 to 2015.

Loss of lock percentage under phase scintillation along with the elevation and the azimuth are shown in Figure 7 to further illustrate the relationship between the loss of lock occurrence percentage and its spatial distribution. It should be noted that the distinctions of satellite orbit densities can also contribute to the unique distribution of loss of lock occurrence percentage in spatial regions, to distinguish this influence from the above results; the spatial diversity of satellite orbital positions was also computed in accordance to different spatial regions, as the red dotted lines show in Figure 7. In this experiment, the elevation interval varies from (30 ± 2.5)° to (90 ± 2.5)° in steps of 5°, while the azimuth varies from (0 ± 15)° to (330 ± 15)° in steps of 30°. Most of the loss of lock cases falls into the region of elevations within 40° to 45° and around 80°, and azimuths within 300° and 330°, 0° and 30°, which is not clearly related to the distribution of satellite orbital positions. This result also proved that loss of lock in different spatial regions varies significantly over the analyzed period.

Comparing the height of the bars, it can be obviously seen that the loss of lock occurrence percentage changes gradually as the year passes by, indicating that the distribution of the loss of lock occurrence percentage under scintillation is not invariant. To be precise, for elevation, the loss of lock occurrence percentage decreased gradually from 40° to 50° and increased from 75° to 85° year by year. For azimuth, the loss of lock occurrence percentage decreased at 30° and increased at 120°. These gradual changes represent that the intensity of active regions shifts between years, which is consistent with Figure 6.

### 3.5. Geomagnetic Storm and Its Effect on Loss of Lock under Ionosphere Scintillation

**Figure 8:**
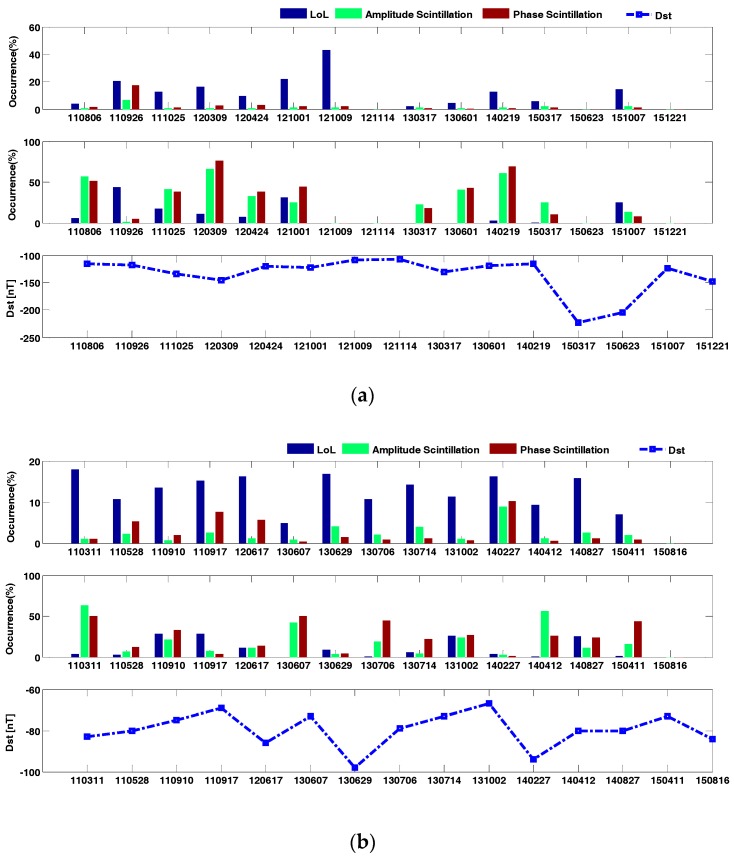
Loss of lock percentage towards geomagnetic storm group A (**a**) geomagnetic storm group B (**b**) from 2011 to 2015.

It should be noted that geomagnetic activities also have an impact on scintillation, as previous studies by Wood and Lima have shown [31,32]. For high latitude regions, local geomagnetic disturbance was correlated with phase scintillation, however for low latitude and mid-latitude regions, geomagnetic disturbances can both deteriorate or depress amplitude and phase scintillation, thus the geomagnetic disturbance on loss of lock occurrence percentage has a complex mechanism under discussion. Generally the Dst index can indicate global geomagnetic status, and is used to determine geomagnetic storms. Carter also calculated the time difference of Dst index to judge the geomagnetic storm phase and its recovery phase [33]. In this experiment, about 30 geomagnetic storms during this solar cycle were selected, from 2011 to 2015. The extent of geomagnetic storms was distinguished by Dst index, with Dst below −100 nT being considered as strong storm, and Dst above −100 nT as a weak or moderate storm, as Table 2 shown. This grouping criterion was just to verify impacts of geomagnetic storm on loss of lock occurrence percentage under amplitude scintillation and phase scintillation. The results are shown in Figure 8a for the geomagnetic storm group A (Dst ≤ −100), and Figure 8b for geomagnetic group B (Dst > −100). In each figure, the upper subfigure was all day scintillation conditions, and the lower subfigure was daytime scintillation conditions. Clear differences were found during geomagnetic storm group A, for the 2012/10/09 storm case, with a relatively high Dst index in group A, and no daytime scintillation was found, whereas in all day scintillation conditions the loss of lock occurrence percentage reached peak value among all storm days in group A. Another distinct feature in group B was the 2013/06/29 case, when just after solstice time, the Dst index went down to the most significant value among group B, and daytime phase scintillation achieved a minimum value, while loss of lock occurrence percentage was relatively high. This indicates that loss of lock occurrence percentage does have a certain correlation with geomagnetic storms, but the linking mechanism is complex to study, due to the contribution of yearly changes of loss of lock characteristics, seasonal and solar activity influences.

**Figure 9:**
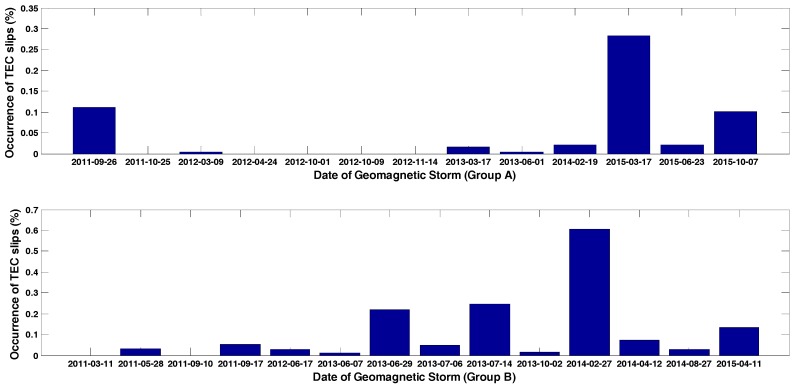
TEC slips towards geomagnetic storm group A geomagnetic storm group B from 2011 to 2015.

The high intensity of loss of lock under storm situations both in group A and group B can be also due to TEC disturbances during geomagnetic storms, which has been considered as one of the criteria to determine loss of lock. To investigate this, the occurrence of TEC slip defined in [34] was calculated for days in both two storm groups, as Figure 9 demonstrates. The 17 March 2015 storm has the most dominant TEC slip, reaching a maximum of 0.3% of all day’s GNSS observations, however TEC slips even don’t happen during a strong storm, as the 9 October 2012, 25 October 2011 and 14 November 2012 cases show, which indicates that geomagnetic disturbances are not directly linked with TEC slips, as a criterion to judge loss of lock. Another finding also properly supports this conclusion, referring to the case in 27 February 2014 storm, when the geomagnetic disturbance index was not most dominant, but the occurrence rate of TEC slip reached to 0.6%, the highest value among all cases in this study. The phenomenon can be due to the high solar activity, but the coupling mechanism still remains complex and worthy of further investigation. It should be mentioned that for group B cases occurrence of TEC slips were higher in FMA and MJJ seasons, but merely found in NDJ season, which is in consistent with the previous study described in Section 3.2.

## 4. Conclusions

In this paper, the temporal and spatial statistical characteristics of loss of lock under ionosphere scintillation observed at Weipa station have been studied with data provided by SWS from 2011 to 2015. Based on the study, the general conclusions for monthly and seasonally loss of lock have been investigated, indicating that loss of lock characteristics at low latitudes of Australia follows specific patterns. These results are of great significance for future studies on GNSS loss of lock features in wider regions.

A general picture of loss of lock event by ionosphere scintillation has been first analyzed to demonstrate the intensity of loss of lock activities in different month and year. Loss of lock events have been proved to be closely correlated with strong phase scintillation, and the average number was 267 cases per month, for an average 72.13% of total month counts. The seasonal variation of loss of lock events starts increasing at 2011 and goes down at 2015, and the most frequent loss of lock events have been discovered to occur during equinox months. It can be concluded that the seasonal variation of loss of lock percentage has an obvious pattern in accordance with local time. The occurrence local time “band” of seasonal loss of lock percentage moved from FMA, to MJJ and ASO. No apparent local time “band” has been found at NDJ, which are just summer solstice months at south-hemisphere. Furthermore, the loss of lock percentage higher than 20% at NDJ was also relatively low, only about 6.9% of the total amount.

Loss of lock percentage under daytime scintillation has been further analyzed. It can be concluded that local time of loss of lock percentage shifted monthly with a clear pattern, and become more severe during solar maximum years (2013 and 2014 in this study). Differently from the general monthly variation, loss of lock occurrence percentage under daytime scintillation was both determined by daytime amplitude scintillation and daytime phase scintillation, and was less correlated with solar SSN index and geomagnetic Kp index. However, a sudden increase of correlation coefficient between loss of lock percentage, SSN and Kp occurred at February 2013, and the lowest values of correlation between loss of lock percentage and daytime amplitude scintillation have been discovered at May 2011 and August 2014, which are both the starting month of the winter solstice and spring equinox in the south hemisphere. Generally, the correlation between loss of lock percentage and daytime phase scintillation index remained stable for most months. This is in accordance with previous studies about the temporal statistics of loss of lock event under phase scintillation.

Another important factor in this study is the spatial characteristics of loss of lock percentage caused by scintillation. The loss of lock percentage in different regions has been analyzed. The most active region of loss of lock percentage changed by year, and reached maximum values at 2014, in consistence with solar maximum. The result is vital compared with satellite orbit value, as guidance for GNSS applications.

Loss of lock percentage in geomagnetic storm periods has been discussed. In this research, around 30 geomagnetic storm cases reported by the literature during this solar cycle have been considered. The intensity of geomagnetic storms was divided by the Dst index into two groups. It is found that some severe geomagnetic storms can lead to large daily losses of lock percentage, but not in all the cases. Daytime loss of lock percentage and daytime scintillation have been also discovered in most geomagnetic storm phases, but in some cases daytime loss of lock percentage and daytime scintillation vanished, the complex mechanism of loss of lock percentage and geomagnetic storm requires further investigation. However, generally the correlation between loss of lock percentage and geomagnetic storms is indirectly seen through this research. This is reasonable according to the most recent studies of scintillation under geomagnetic storm conditions, with evidence that geomagnetic storms can both deteriorate and depress ionosphere scintillation, even with no influence on ionosphere scintillation.

Further studies will concentrate on the loss of lock features under global data sets and the theoretical mechanism of the relationship between local time and the loss of lock in equatorial and low latitude regions, based on the conclusions drawn in this study. Additionally, statistical studies of loss of lock by combining factors more than scintillation can also be considered as one of the key issues for further study.

## Figures and Tables

**Table 1:** The parameters provided by SWS GPS ISMs observation files.

Parameters Contained in Files	Definition
Time	The data record universal time, in decimal hours
PRN	Pseudo-Random Number of the GPS satellite
Azimuth angle	Azimuth angle of the GPS satellite
Elevation angle	Elevation angle of the GPS satellite
L1 Carrier to Noise Ratio	*CN*0 of the signal measured on the *L*1 frequency
Amplitude Scintillation Index *S*_4_	The raw “*S*_4_” amplitude scintillation index
*S*_4_ Index Correction(*S*_4_Corr)	The estimated error to raw *S*_4_ due to internal receiver noise
FINAL *S*_4_ Index (Final_*S*_4_)	Final_*S*_4_ = *S*_4_ − *S*_4_Corr
Phase Scintillation Index (Sigma60)	The raw observed phase scintillation, calculated by the standard deviation of the carrier phase over 1 min

**Table 2:** Geomagnetic storms from 2011 to 2015 in solar cycle 24.

Group A Strong Storm Cases (Dst ≤ −100 nT)		Group B Weak Storm Cases (Dst > −100 nT)	
date	Dst (nT)	date	Dst (nT)
20110806	−115	20110311	−83
20110926	−118	20110528	−80
20111025	−134	20110910	−75
20120309	−145	20110917	−69
20120424	−120	20120617	−86
20121001	−122	20130607	−73
20121009	−109	20130629	−98
20121114	−108	20130706	−79
20130317	−131	20130714	−73
20130601	−119	20131002	−67
20140219	−116	20140227	−94
20150317	−223	20140412	−80
20150623	−204	20140827	−80
20151007	−124	20150411	−73
20151221	−148	20150816	−84
